# Non-Invasive Cattle Body Temperature Measurement Using Infrared Thermography and Auxiliary Sensors

**DOI:** 10.3390/s21072425

**Published:** 2021-04-01

**Authors:** Fu-Kang Wang, Ju-Yin Shih, Pin-Hsun Juan, Ya-Chi Su, Yu-Chieh Wang

**Affiliations:** Department of Electrical Engineering, National Sun Yat-sen University, Kaohsiung 80424, Taiwan; fkw@mail.ee.nsysu.edu.tw (F.-K.W.); d093010002@student.nsysu.edu.tw (J.-Y.S.); m063010103@student.nsysu.edu.tw (Y.-C.S.); m073010106@student.nsysu.edu.tw (Y.-C.W.)

**Keywords:** core body temperature, environmental factors, infrared thermography (IRT), noninvasive, multiple sensors architecture

## Abstract

To achieve a sensitive and accurate method in body temperature measurement of cattle, this study explores the uses of infrared thermography (IRT), an anemometer, and a humiture meter as a multiple sensors architecture. The influence of environmental factors on IRT, such as wind speed, ambient temperature, and humidity, was considered. The proposed signal processes removed the IRT frames affected by air flow, and also eliminated the IRT frames affected by random body movement of cattle using the frame difference method. In addition, the proposed calibration method reduced the impact of ambient temperature and humidity on IRT results, thereby increasing the accuracy of IRT temperature. The difference of mean value and standard deviation value between recorded rectal reference temperature and IRT temperature were 0.04 °C and 0.10 °C, respectively, and the proposed system substantially improved the measurement consistency of the IRT temperature and reference on cattle body temperature. Moreover, with a relatively small IRT image sensor, the combination of multiple sensors architecture and proper data processing still achieved good temperature accuracy. The result of the root-mean-square error (RMSE) was 0.74 °C, which is quite close to the accurate result of the IRT measurement.

## 1. Introduction

Mammals remain healthy when their body temperature remains constant with an effective cooling system. However, as climate change has significantly caused the rise of environmental temperature, a temperature that is too high may affect the amount and quality of milk and beef in cattle. In the worst scenario temperature might even cause the death of cattle [1]. An extreme case was the heat wave that occurred in July 1995. The event led to the death of approximately 3750 cattle. Estimates of direct loss was 2.8 million US dollars and production loss was 28 million US dollars [2].

There are three common underlying diseases of cattle, and the cause of diseases is usually reflected in body temperature. First, bovine mastitis has a negative effect on milk production and quality, which will cause significant economic problems [3,4,5,6]. Cattle inflicted with mastitis would have an average body temperature 2.64 °C higher than healthy cattle [7]. Second, the bovine respiratory disease complex (BRDc) is a complex of diseases characterized by many types of bacterial infection. BRDc is the most common and costly disease of feedlot cattle in the world. One of the common symptoms of pneumonia caused by BRDc is fever in excess of 40 °C [8,9], a fatal condition. According to references [10], most livestock in feedlots did not show traditional clinical symptoms of BRDc. Over 50% of the calves were not confirmed cases. Usually, four days after contraction the disease was confirmed, and therefore early treatment of BRDc on calves could not be applied.

Third, heat stress can be defined as physiological stress experienced as a result of excessive heat [11]. Cattle that suffer heat stress obviously are affected in terms of milk production and fertility [12,13]. In [14], Zimbelman et al. suggested that the best method to assess heat stress is to measure rectal temperature greater than 39 °C and respiration rates greater than 60 beat/min. Although confirmation and early diagnosis of diseases requires clinical criteria, the diagnosis of various diseases still needs body temperature or physiological parameters. Compared to other internal parameters such as blood and respiration, body temperature has been considered for a long time as a criteria for measurement and judgment of animal well-being and disease by veterinarians and owners. Now, benefiting from the advance of technology and instruments, and also from in-depth research on cattle behavior and temperature models conducted by researchers [1,15], an important new research direction has invested in the long-term monitoring of cattle core temperature for the wellbeing of livestock and avoiding economic loss.

To detect the core body temperature of animals, commonly, the use of thermometers employs thermistors. A thermistor is a semiconductor made of metal oxide. The resistance of this semiconductor varies with temperature sensitively. The best operating range is between 0–200 °C. However, a thermistor is also a non-linear component, the correct reading of which requires a lookup table established in a control unit. Traditionally, body core temperature can be easily obtained with a rectal thermometer, but to acquire body temperature cattle need to be restrained. This is likely to cause a stress response, resulting in stress-induced hyperthermia that subsequently decreases the accuracy of body temperature assessment [16]. In short, the traditional method for rectal temperature assessment may not be suitable for long-term assessment. Contact sensors are more or less invasive. They are subcutaneously injected or implanted under muscle tissue. Common contact methods require thermometers to be placed in the rectum [17], ear canal [18], or vagina [18,19]. In practice, the thermometer should be swallowed, but the limitation of the digestive system may affect assessment result, which, in addition, would be affected by the temperature or size of the cattle [19,20], and a cold floor on which animals lie [21]. Assessment results are better when correlated to temperature acquired by thermometers in contact with the ear canal and eardrum, and to rectal temperature acquired by intravaginal sensors (sensitivity to tiny variations is also improved) [19,20]. Nonetheless, risk of infection might be increased when sensors are placed at mucosa or muscle tissue [18].

Previous sensors that need direct contact with tissue to measure temperature are not suitable for long-term monitoring. That is why we adopt infrared (IR) for our sensors. The use of IR technology can remotely measure temperature. The principle of the infrared thermography (IRT) is to image the infrared radiation emitted from the object if the temperature is above absolute zero. This means only the surface temperature is recorded. In a biology-related temperature, the radiation reaches its peak at an IR wavelength of 8–12 μm [22]. Since 2005, IRT has been applied by veterinarians and animal researchers to detect surface temperature [23,24], but the problem of thermography is to determine the area where the pictures should be taken [5]. The IRT is useful for early detection of laminitis or leg injuries in cattle or horses. Alsaaod et al. detected laminitis in dairy cows by measuring the temperature difference between healthy and affected feet [25]. Poikalainen et al. [26] and Nikkhah et al. [27] used IR in cattle to detect injuries on legs or other body parts. The eye temperature measured with the IRT can be used for detecting an acute response of stress or pain of cattle [24,28,29]. In cattle, the surface temperature of udder rises when clinical mastitis occurs [6], and this variation can be detected with the IRT in a non-invasive way [4].

Recently, IRT not only provides the body temperature of the cattle but also can be used for other detections. Lowe et al. validated the use of IRT to measure the respiration rate as an alternative method. From the thermal image, temperature changes at the nostrils during inhalation and exhalation [30]. Yáñez-Pizaña et al. monitored the temperature difference of the weaned piglet on its ocular area due to the effect of environmental enrichment [31]. For the aforementioned disease of cattle, Giro et al. monitored the behavior of beef cattle using a portable IRT, and they found that cattle tend to use shade or water to avoid heat stress [32]. Moreover, early detection of bovine mastitis by IRT is a known technique. Tangorra et al. inspected the temperature changes during the milking process at the cow udder teat [33]. Zaninelli et al. proposed a new location of the thermal image to aim at and its temperature was in a significant relationship with somatic cell count [34]. Zhang et al. proposed an algorithm which can be used to detect the eyes and udders of dairy cows, and automatically identify cow mastitis with accuracy of 83.3% [35]. These applications that use IRT to obtain temperature may not require precise surface temperature, but they are indeed limited by environmental influences.

An infrared sensor displays the surface temperature based on the total radiation heat, but external factors might affect real temperature and, therefore, corrections are required. Nowadays software in infrared devices provide modifiable parameters to mitigate the effect from external factors. Factors that affect the IRT to detect heat radiation can be categorized in three types: 1. Reflection, a common problem in electronic devices, can be compensated if the exact reflection coefficient is given; 2. Emissivity, the ability of different materials to emit infrared energy, may be widely different and should be adjusted; and 3. Environment, including moisture and temperature, may also be considered to calibrate the instrument. Most references applying the IRT suggested environmental factors might affect the IRT measurement result on site [24,36], and several other references discussed the effects of environmental factors. Gloster et al. [37] took thermal images of cattle’s hooves at different ambient temperature. The result showed that the ambient temperature influenced the accuracy and validity of the infrared readings measured by the IRT. The variability in hoof temperature was greatest at lower ambient temperature. McCafferty et al. claimed that heat radiation readings might not be taken in to account under the condition of low solar radiation; however, as the sunshine was sufficient and the spot of exposure changed the conductivity and emissivity, the IRT measurements were affected by short-wave reflection [23]. Chruch et al. found that the influence of humidity was small at a distance of 1 m, but became significant at greater distances and higher temperature. Moreover, the measurement results showed that a temperature error of 0.78 °C occurred at a wind speed of 3.3 m/s; direct solar loading compared to that on the other eye measured in shade had a 0.6 °C error within a 30-min time frame, proving that air flow and solar loading have a direct effect on surface temperature [38]. Dragomir et al. evaluated IRT temperature on different emissivity values with a copper bar covered with several types and thicknesses of dust and an electrical tape with an emissivity of 0.96 close to that of the skin as the reference. The result showed that the types of dust did not affect IRT temperature but emissivity [39].

**Figure 1:**
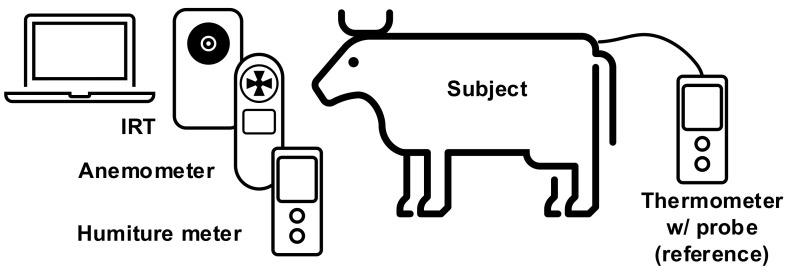
Experimental setup of the proposed system architecture.

Currently, the technique of IRT body temperature sensing neither exclude environmental factors nor cattle movement. To acquire accurate cattle core temperature and considering all the aforementioned problems, this study proposes an IRT sensing technique which combines an anemometer and humiture meter as the auxiliary sensors. These multiple sensors architecture provide accurate temperature readings. As in Figure 1, the experiment targeted the eye socket of cattle with the IRT. In the process we also detected wind speed, temperature, and humidity as reference for subsequent data processing. The reference signal was the body temperature. In Section 2, we gather environmental factors, as shown in Figure 1, and the experimental setup recorded data using three devices and different functionalities. In Section 3, the proposed signal processing flow removes the effects of wind and cattle movement, and after that the calibration method is utilized. Section 4 shows measurement results as demonstrated and discussed, and Section 5 of this study is the conclusion.

## 2. Experimental Setup

**Figure 2:**
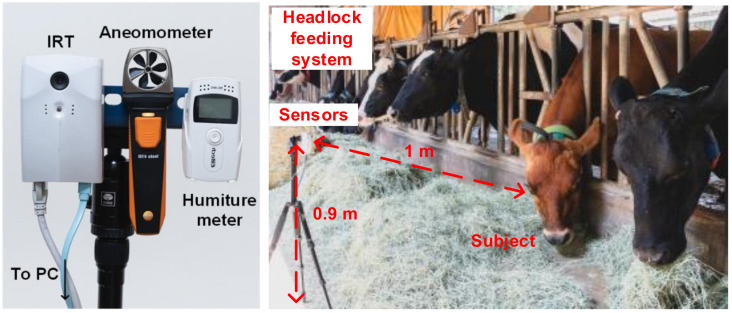
Photography of environment in pasture while recording the data.

The multiple sensors architecture experiment setup is portrayed in Figure 2. The IRT, anemometer, and humiture meter were installed facing the cattle in the same direction. A computer was used to collect readings sent back from the sensors. Cattle core temperature was recorded using a thermometer with a probe as a reference for measuring error. As shown in Figure 2, the sensor was placed in front of the feedlot, and the necks of the cows were restrained by a headlock feeding system to ensure the cattle were stationary and their heads were not moving excessively during the measurement. The experiment was conducted in a covert area, so the surface temperature of the cattle was not affected by the sun [38]. Considering the factory default calibration setting and field of view of the Lepton, the IRT, anemometer, and humiture meter were all horizontally installed on the same tripod. The sensor was installed at a place with a height of 0.9 m and a distance of 1 m from the cattle. The experiment was conducted during the feeding time of the cattle, which was 10:00 AM to 02:30 PM every day, and the ambient temperature was 14–29 °C. At the same time, the ranch technician put a thermometer inside the anus of the cattle with a depth of 10 cm to record the rectal temperature. The breeds were Holstein and Jersey, readings were recorded repetitively 107 times. Considering the subsequent IRT signal processing, some of the environmental factors and body movements were deleted, and to ensure an adequate number of data were recorded and to reduce the effect of noises, each set of data took 90 s, which included IRT temperature, rectal temperature, wind speed, environmental temperature, and environmental humidity. Such a setup also considered the three types of factor that affected the IRT temperature. For reflection, the adopted IRT device was calibrated inside the factory, and the control unit of the device could accurately record the voltage level. For emissivity, the IRT lens was kept 1 m from the subjects and targeted at the heads, and without altering the materials and light exposure, the emissivity was set to 0.95. For the environmental factor, this was excluded to reduce negative influences on the IRT temperature.

**Figure 3:**
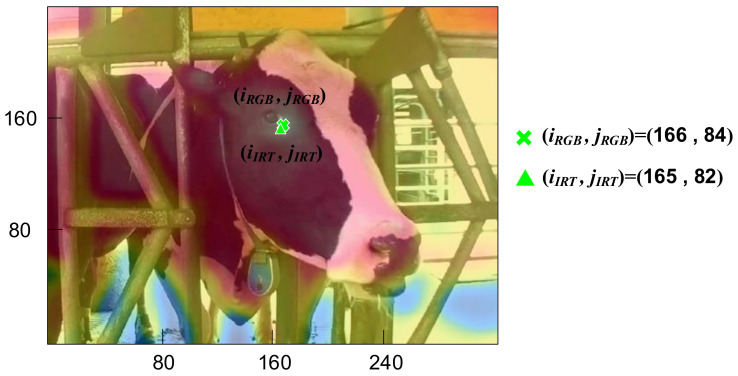
Optical image and infrared thermography fused in one picture.

Our IRT device, the AD-HF048 manufactured by ADE, consists of a thermal image sensor and an optical image sensor. The former is the Lepton manufactured by FLIR with a resolution of 80 × 60-pixel, and fields of view (FOV) of 51° (horizontal) and 38.25° (vertical). Due to export control, the frames per second (FPS) is limited to 7. When temperature is between 30–45 °C, allowing the accuracy to be ±0.75 °C, and the thermal sensitivity is less than 0.05 °C according to the manufacture datasheet [40]. For the convenience of observation, a 200-megapixel camera, with a FOV of 65° (horizontal) and 40° (vertical), was included in our setup to cohere with the FOV of the IRT. Optical devices were not required during the experiment. Indicated in Figure 3, optical images and IRT images were overlayed to determine that the cattle’s lacrimal glands matched the location of the highest surface temperature. As the resolutions of the two sets of images were both 320 × 40 pixels, *(i_IRT_, j_IRT_)* and *(i_RGB_, j_RGB_)*, locations of the lacrimal gland, and (165, 82) and (166, 84), locations of the highest surface temperature, were almost perfectly overlayed. The anemometer in the experiment was 410i manufacture by TESTO, which records wind speed per second and is connected to other devices through Bluetooth. The sensing range of 410i is 0.4–30 m/s, with an accuracy of ±0.2 m/s and a resolution of 0.1 m/s. The humiture meter in the experiment was an RC-4HA manufactured by Elitech, which records data through USB connection. Every 10 s the meter records data including environmental humidity and temperature. The sensing range of environmental temperature is −30–+60 °C. The accuracy is ±0.5 °C and resolution 0.1 °C under the range between −20 – +40 °C. The environmental humidity range is 0–99% RH. The accuracy is ±3% RH and resolution 0.1% RH under 25 °C and 20–90% RH. The thermometer in the experiment was an RC-4HC manufactured by Elitech. The sensing specification of RC-4HC is identical to that of the RC-4HA. The difference is that RC-4HC includes a waterproof probe, with a sensing range between –40–+85 °C. In this research, all the hardware of the multiple sensors architecture is listed in Table 1.

## 3. Signal Processing

**Figure 4:**
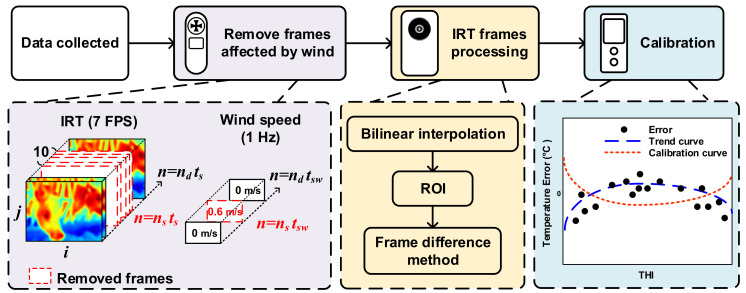
Block diagram of the proposed work.

**Figure 5:**
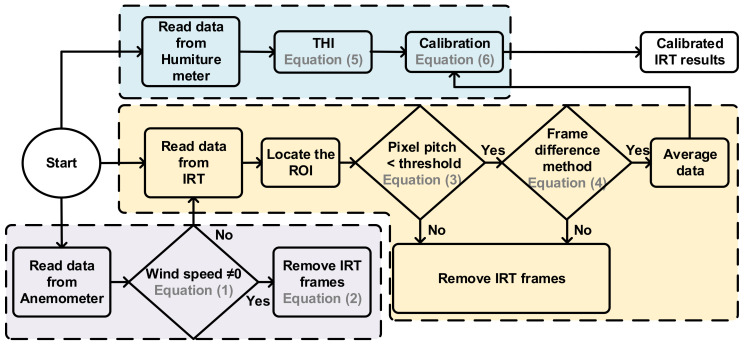
Signal processing flow of the proposed multiple sensors architecture.

The block diagram of this research is shown in Figure 4, which indicates the removed frames because of wind, IRT frame processing, and temperature calibration. The detailed information of the signal processing flow is shown in Figure 5. The auxiliary sensors cooperated with IRT and provide a decision to prune IRT frames which were affected by the environment. These decisions will be described below.

**Figure 6:**
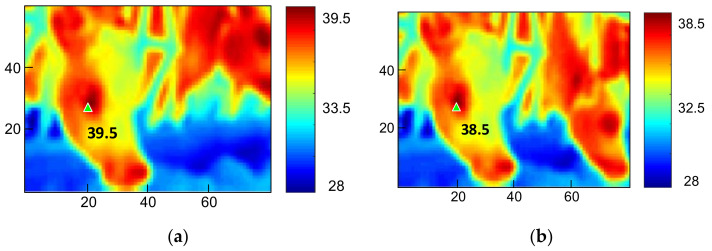
The effect of wind speed on IRT. (**a**) The lacrimal gland temperature is 39.5 °C in the absence of wind. (**b**) The lacrimal gland temperature is 38.5 °C at wind speed of 0.6 m/s.

A known fact is that surface heat dissipation as a result of air flow could significantly affect the temperature IRT. Figure 6a shows a windless condition, and the highest temperature, 39.5 °C, in the image was in the lacrimal gland. Figure 6b shows a result under a wind speed of 0.6 m/s, and the temperature in the lacrimal gland was 38.5 °C, a 1 °C difference from the windless result. Signal processing first removed the frames affected by wind speed. Wind speed was designated by *ws*[*n*], and the IRT temperature function by *T*[*ROI,n*]. *ROI* is the region of interest (ROI) in IRT frames. This research selected frames higher than 37 °C as the ROI, which was designated as a two-dimensional coordinate [*i*, *j*], and was the ith row and jth column in each frame. Discrete time was designated as *n = n_d_t_s_*, where *n_d_* was the number discrete data entries. *t_s_* = 1/7 was the sampling cycle of the IRT, and *t_sw_ =* 1 was the sampling cycle of the anemometer. As shown in Figure 4, data were synchronized to solve the discrepancy between various sampling rates in different devices, and numerical sequences with lower sampling rates went through an upsampling process. Thus, the equivalent sampling cycle of the anemometer was set to *t_s_*. Assuming that wind speed is not 0 as *n = n_s_t_s_*, the discriminant is:

(1) $$ws[n]\left|{}_{n=n_{s}t_{s}}\right.\neq 0$$

As Equation (1) is true, time *n = n_s_t_s_* is discarded. In addition, surface temperature needs response time to recover and, therefore, the recovery time was set to a margin of 3 frames, meaning that 10 frames, *n_d_ = n_s_* − *n_s_ + 3*, were discarded. The equation for signal processing is represented as:

(2) $$T[ROI,n]\left|{}_{n_{d}=[n_{s},n_{s}+10]}\right.=[\;],$$

A method not only discards data affected by wind speed, but also reduces useless information to increase computing efficiency.

**Figure 7:**
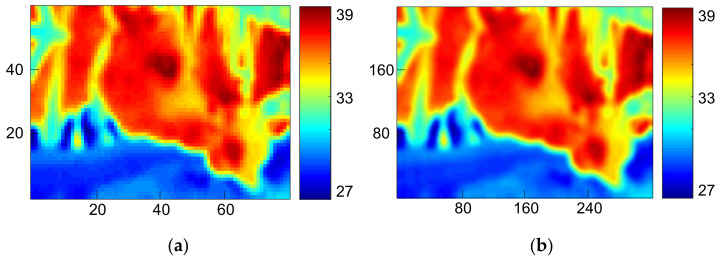
IRT frame processing. (**a**) Original IRT picture. (**b**) After the bilinear interpolation.

Next, IRT frames were processed. The procedures included image enhancement and re-adjustment of color maps for the distribution of color scales for easier observation. Then all frames underwent resampling with bilinear interpolation to solve the raster images caused by insufficient resolution at edges. As shown in Figure 7a, a new pixel was calculated by the weighted average of attributes of four-pixel pitches from the point to be interpolated. Such a method is used to acquire a larger image. As indicated in Figure 7b, the resolution was enlarged from 80 × 60 pixels to an equivalence of 320 × 240 pixels. Although a bigger-sized image sensor can obviously obtain images with higher resolution, we still used a smaller-sized one for better accessibility and lower costs. Interpolation can enhance resolution and improve the subsequent ROI determination and frame difference method, but the contour may be blurred due to the low-pass filtering effect. This research, however, does not require a clear contour for object recognition, and that is why a trade-off was inevitable.

**Figure 8:**
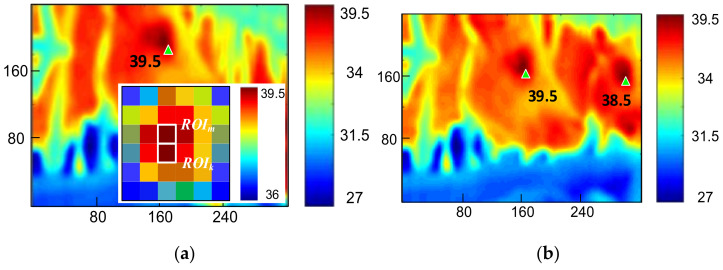
Region of interest in each frame. (**a**) A frame with only one cow. (**b**) A frame with two cows.

In previous sections, we have discussed that the highest-temperature spot in an IRT image is the eye socket; however, as indicated by experimental results, the determination might be confused if adjacent bodies of cattle moved inside the IRT FOV even if the lens was aimed at the target cattle. Therefore, we would like to ensure that an image only contains one single object. When multiple ROI images were captured, distances between the images were calculated to determine whether they satisfied the pixel patch threshold preset by the software. If the threshold was passed, more than one cow was recorded and that frame was removed. Figure 8a is an image containing only one cow, and the snapshot is a 6 × 6 diagram of the highest temperature spot. Two pixels were higher than 37 °C, and they were designated to *ROI_m_* and *ROI_k_*, respectively. The cause of such a phenomenon was the projection angle of the reception of IR and IRT sensors, and the image was dispersed to adjacent pixels. The hardware in Chapter 2 was used with triangle similarity. Assuming the length of the cattle’s eyes being 4 cm, a 4 cm object occupied 4 pixels on the screen captured by the IRT image sensor, which was 1 m from the target. Thus, the pixel pitch threshold was equivalently designated 16 pixels, and the condition was:

(3) $$\left\lceil max\left(\sqrt{{\left(i_{k}-i_{m}\right)}^{2}+{\left(j_{k}-j_{m}\right)}^{2}}\right)\right\rceil \leq 16,$$

**Figure 9:**
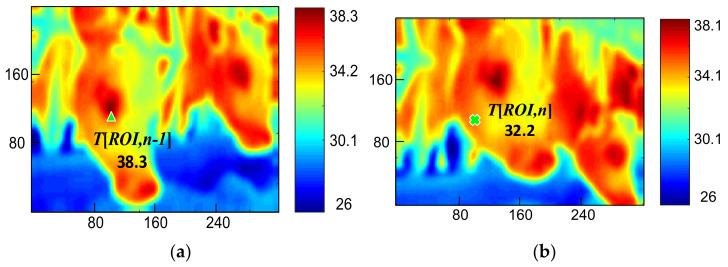
Excessive head movement removal. (**a**) time *n*−1 frame. (**b**) time n frame.

Whether pixel pitches are smaller than the threshold should be determined only if the ROI is greater than 1; if the pixel pitches are greater than the threshold, that frame should be removed. Therefore, Figure 8a does satisfy Equation (3). Figure 8b shows two cows entering the FOV, and pixels higher than 37 °C are marked as green triangles. The resolution of the two pixels pitches is lower than 124 pixels and does not satisfy Equation (3). Nonetheless, IRT images were affected because in the experiment the cattle were eating and the swing of heads might blur images or disrupt airflows. In response to this issue, we adopted the frame difference method to deduct the temperature of two consecutive frames to determine whether the difference fitted the threshold. The discriminant is:

(4) $$\left|T[ROI,n-1]-T[ROI,n]\right|\leq 1$$

where the ROI is designated to the location of frame *n*−1. Figure 9 indicates how one cow moved continuously in an image, and Figure 9a is the detection of frame *n*−1. *T[ROI,n−1]* was 38.3 °C, and *T[ROI,n]* in Figure 9b was 32.2 °C. The temperature difference of the ROI image with stationary cattle should approach 0 °C; otherwise, that frame should be removed. Substituting data in Figure 9 into Equation (4) found that the temperature difference was approximately 6.1 °C, and the frame was removed because the result was over the threshold. The result also indicates that ROI images will be re-defined if the measurement results do not satisfy Equations (1) and (4).

We have eliminated the effect caused by wind speed and body movement to the IRT images, now we continue to calibrate the measurements with the removal of environmental temperature and humidity. The authors of this study live on an island in the subtropical climate, and the average humidity is 70–80% RH. References have indicated that heat dissipation from animals’ body surface is related to relative humidity, environmental temperature, and convection [41]. Moreover, relative humidity is linearly correlated to environmental temperature, and that is why this study calibrated the IRT data with the Temperature-Humidity Index (THI) [42], and the THI is shown in Equation (5):

(5) $$THI=\left(1.8\times T_{db}+32\right)-\left(0.55-0.005\times RH\right)\times \left(1.8\times T_{db}-26\right)$$

where the unit of dry bulb temperature, *T_db_*, is Celsius temperature and represents environmental temperature; *RH* is the relative humidity with a unit %RH.

To narrow down the error between IRT temperature and rectal temperature, we created a diagram of temperature error using the THI, and then the distribution plot of the quadratic trend curve was calculated. This trend curve and the symmetrical curve with no error were the calibration curves, which are a method for comparing the errors between an environmental parameter and a rectal temperature reading. Calibrated results could be provided after the calibration curve and the original data are added.

## 4. Measurement Results and Discussion

**Figure 10:**
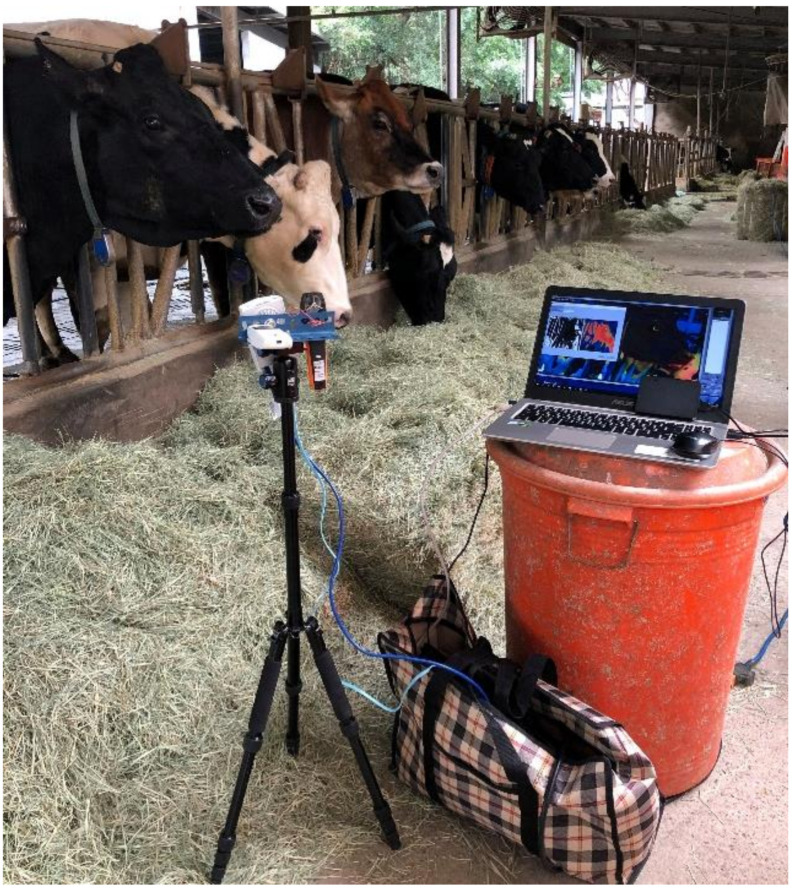
Photography of the experiment for noninvasive cattle body temperature monitoring.

**Figure 11:**
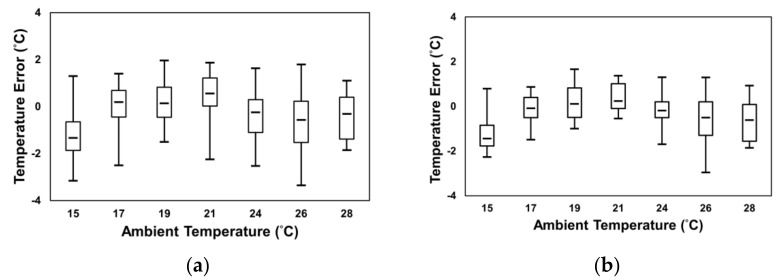
Temperature error versus the ambient temperature (**a**) before (**b**) after IRT frame processing.

**Figure 12:**
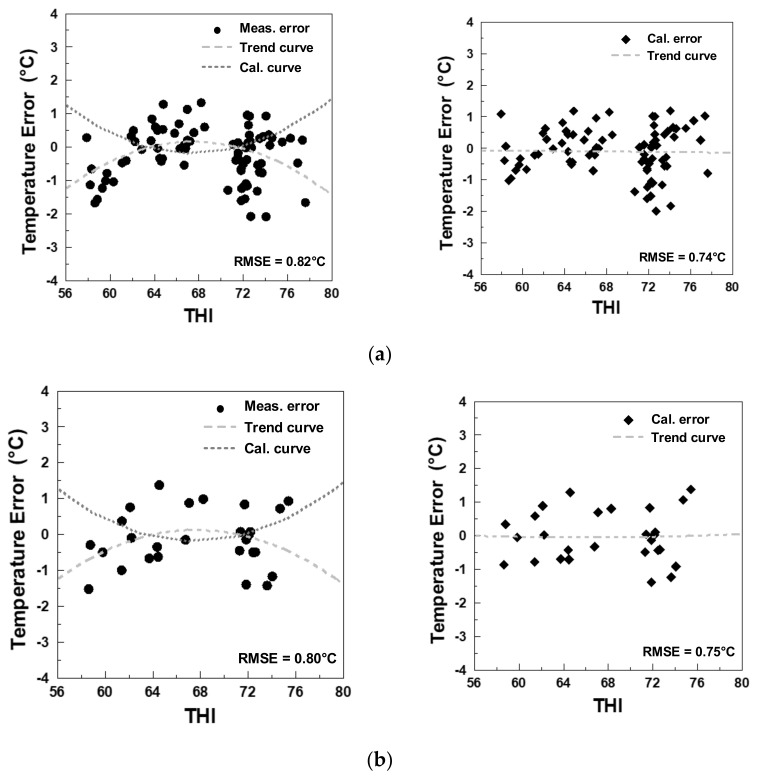
Measured result before and after calibration. (**a**) Training set. (**b**) Testing set.

The photography of the experiment for non-invasive cattle body temperature monitoring is shown in Figure 10, in which you can see those sensors were mounted on a tripod. A PC was connected to sensors via RJ45 cable and Bluetooth while running Matlab. According to the hardware parameters and data analysis methods discussed in previous chapters, the IRT data length was less than 90 s because some data were removed after the IRT frame processing. To determine the correlation between ambient temperature, IRT temperature, and rectal temperature and also to observe how the IRT frame processing improved the measurements, Figure 11 displays temperature errors under different ambient temperature in a box plot. For easier observation, data retrieved by the IRT and reference thermometers were averaged every 10 s. The original errors are shown in Figure 11a, where the temperature error is the difference between IRT temperature on ROI and rectal temperature. After IRT frame processing, the overall errors were significantly reduced, as compared to data in Figure 11b. When the ambient temperature was 21 °C, original data errors were ±2 °C, and the median 0.55 °C. The processed temperature errors were approximately ±1 °C, and the median was 0.235 °C. Furthermore, with the calibration method proposed by this research randomly categorized 107 entries of data to 80 training sets and 27 validation sets to acquire the calibration curve and evaluate the results of calibration. The data before and after the calibration are listed in Figure 12a. The image to the left is the calibrated 80 training sets. The horizontal axis is the THI, and the vertical axis is the temperature error. This scatter plot shows that the THI data are mostly distributed between 72–80, and such distribution was fitted to a quadratic trend curve, where the measured error, calibrated error, trend curve, and calibration curve were represented by circle, diamond, dotted line, and dashed line, respectively. This trend curve is designated Equation (6):

(6) $$Temperature\;error=-0.0105{\left(THI\right)}^{2}+1.4197\left(THI\right)-47.816,$$

**Figure 13:**
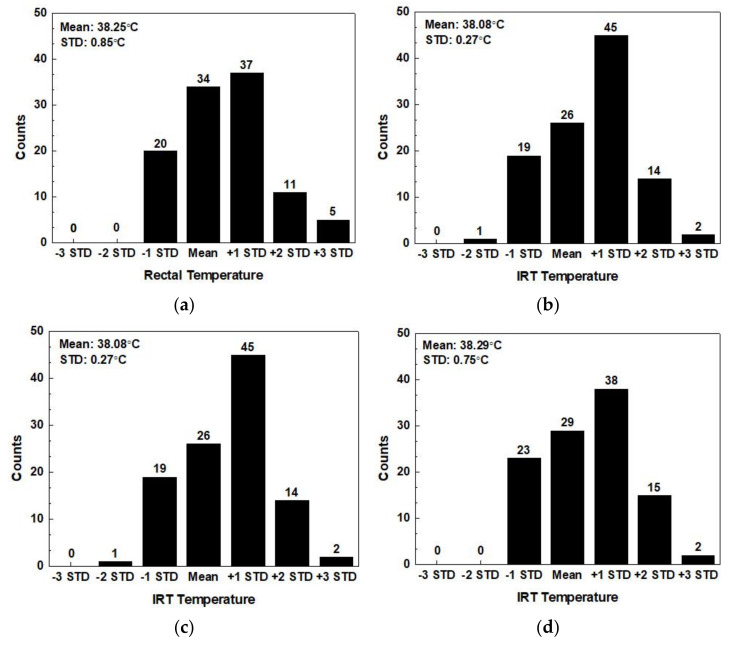
Measurement for IRT eye temperature and rectal temperature on 107 cattle. (**a**) Rectal temperature. (**b**) Original IRT temperature. (**c**) After IRT frame processing. (**d**) After calibration.

Finally, we calculated the average and standard deviation of the results acquired in each procedure. Figure 13 records the distribution of 107 entries of data to find the discrete levels of the readings of the IRT and the rectal thermometer. Figure 13a is the histograms of data distribution of rectal temperature with a thermometer. The mean is 38.25 °C, and the STD 0.85 °C. Figure 13b is the original data distribution using the IRT. The mean is 38.08 °C, and the STD 0.27 °C. Temperature difference exists in the original measurement results. The mean difference of the measurements acquired by the two sensors is 0.17 °C, and the STD difference 0.58 °C. The IRT frame processing is performed on the results of Figure 13b and is recorded as Figure 13c, the results of which shows a mean of 38.08 °C, and an STD of 0.27 °C without changing the original data distribution. The calibrated distribution graph is displayed by Figure 13d. The mean is 38.29 °C, and the STD 0.75 °C. The mean difference from the rectal temperature in Figure 13a is 0.05 °C, and the STD difference is 0.13 °C. In short, the distribution of the IRT is close to that of the rectal thermometer.

Most researchers have studied the correlation between the IRT temperature of individual parts of cattle and the environment. Some on them utilized IRT to detect the diseases. In brief, the researchers relied on the accuracy of the temperature that IRT could provide. Table 2 discussed the comparison of the body temperature using different technologies, several of which were adopted by us including the position aimed at, and the differences of reference body temperature between mean difference and STD difference. The reference body temperature can be rectally or other accepted proxy methods. With the invasive method studied by Lee et al. [43], the button-like digital thermometer was placed subcutaneously. The result measured in upper scapula was considered to be the least influenced by ambient temperature. With the data recorded in the 6 months of winter and 4 months of summer, the results of the mean difference and STD difference were 1.52 °C and 0.69 °C. In [37], Gloster et al. studied the foot-and-mouth disease, and IRT was performed on hooves. Eye temperature was measured as a proxy for body temperature. The mean difference between rectal temperature and IRT temperature on eyes were about 2 °C, consistent with the result in Figure 12a. In the study of Salles et al. [44], the temperature of a multiple body region was recorded using the IRT. Under the ambient temperature of 22.5 ± 4.8 °C and humidity of 75.1 ± 15.8% RH, as shown in Table 2, the mean difference and STD difference were 1.14 °C and 0.16 °C, respectively. Though the multiple areas of temperature were recorded, the mean difference between rectal temperature and IRT eye temperature were the lowest. In [45], Giro et al. evaluated the use of microchip thermometer and IRT in monitoring the body temperature of beef cattle. Under the ambient temperature ranging from 17.2 °C to 29.3 °C and humidity from 46.2% RH to 100% RH, the mean difference and STD differences were 1.43 °C and 0.5 °C using IRT temperature on eyes; the mean difference and STD differences were 5.51 °C and 1.42 °C using IRT temperature on ears. A study [46] evaluated the heat tolerance of five zebu breeds using hematological and thermographic response, the mean and STD differences were calculated with eye temperature and core temperature, and the results were 3.04 °C and 0.32 °C, respectively. Compared to [44,45], ref. [46] showed greater errors because of the following parameters: 1. sensors were farther to the object; 2. the experimental site, where the ambient temperature difference and humidity being 24.4 °C and 63% RH, showed significant change in environmental factors; and 3. the average wind speed at the site was 1.6 m/s and the highest wind speed 7.7 m/s.

Compare to [47], the proposed method improved mean difference and STD difference, which means the prediction of core temperature more accurately. Furthermore, it would be possible to analyze the factors causing temperature errors and consistency of IRT and reference temperature if the RMSE is provided. Compared to the aforementioned literature, the next two considered environmental factors are wind and solar radiation. In [38], the study focused on how the environmental factors such as wind speed and solar loading affected the IRT. The equipment was installed within a barn to reduce the environmental effects of wind and solar radiation. Mean difference and STD difference were 1.69 °C and 0.21 °C. The study of Hoffman et al. investigated the body temperature by measuring different regions of cattle, such as eye, ear, and vulva [47]. The filmed regions were selected carefully to ensure they were not exposed to direct sunlight nor wind. The mean difference and STD difference were 1.69 °C and 0.21 °C. Moreover, the software (software PI Connect 2.0.2009.0) they used can input the ambient temperature to analyse the temperature of infrared images, and it could be regarded as a concept of sensor fusion. Benefiting from the effect of multiple sensor information, although the resolution of the sensor was relatively small, the software still achieved better performance compared with other literature in Table 2. In the current study, the effect of sensor fusion provides additional information to remove the unwanted frames and to compensate for the influence of environmental factors. With the help of a multiple sensors architecture, the result of the RMSE achieved a hardware limitation. Compared to [47], the proposed method improved mean difference and STD difference, and proves that the prediction of core temperature more accurate. Furthermore, it would be possible to analyze which factors are causing temperature errors and to improve the consistency of IRT and reference temperature if the RMSE is provided.

Future work will firstly focus on the situation when cattle is not constrained by the headlock feeding system, which means eye recognition should be more accurate, and this concept can be achieved by referring to the study of Lowe et al. [48]. Second, the effect of air flow should be considered rather than removed from the frame, which will maintain the FPS. Third, referring to the study of Cuthbertson et al., the higher FPS of the IRT can reduce the length of time it takes to collect IRT data [49].

## 5. Conclusions

This work proposed a IRT sensors fusion architecture for non-contact cattle body temperature measurement. Since the performance was in good agreement to the reference, this system can be used as an alternative to the traditional method which is not suitable for long-term monitoring. With the help of an anemometer and humiture meter, the frames affected by wind speed were removed and the influence of environmental factor was calibrated. Furthermore, the frame difference method detected the random body movement of cattle and located the ROI in each frame. After the calibration for THI, the RMSE reduced to 0.74 °C, and pushed the accuracy to its hardware limitation. Moreover, the difference of mean and the STD between rectally recorded reference temperature and IRT temperature is 0.04 °C and 0.10 °C, respectively. To the best of the authors’ knowledge, the proposed system had the lowest mean difference and STD difference of the currently proposed method.

The following conclusions were drawn:

(1) The proposed non-invasive core body temperature method can be an alternative to traditional invasive method.
(2) The environmental factor such as air flow and humidity were considered.
(3) The proposed system had lowest mean difference and STD difference of the currently proposed method.

## Figures and Tables

**Table 1:** Sensors and specifications.

Sensors	Measuring Range	Accuracy	Sensitivity	Logging
IRT	30–45 °C	±0.75 °C	0.05 °C	7 FPS
Anemometer	0.4–30 m/s	±0.2 m/s	0.1 m/s	1 Hz
Humiture meter	20–90% RH	±3% RH	0.1% RH	0.1 Hz
Thermometer	−20 – +40 °C	±0.5 °C	0.1 °C	0.1 Hz

**Table 2:** Comparison of the measurement of cattle’s body temperature using different technologies.

Reference	Technique	Position	Resolution	Distance (m)	Multiple Sensors	Difference (°C)	
						Mean	STD
[43]	Thermometer	Upper Scapula	0.5 °C	-	No	1.52	0.69
[37]	IRT	Eye	160 × 120 pixels	1–2	No	2	-
[44]	IRT	Eye	128 × 96 pixels	0.2	No	1.14	0.16
[45]	IRT	Eye	640 × 480 pixels	0.5	No	1.43	0.5
[46]	IRT	Eye	320 × 240 pixels	1.5	No	3.04	0.32
[38]	IRT	Eye	320 × 240 pixels	1	No	1.69	0.21
[47]	IRT	Eye	160 × 120 pixels	1	Yes	1.82	0.72
This work	IRT	Eye	80 × 60 pixels	1	Yes	0.04	0.10

## Data Availability

All calculated and measured data will be provided upon request to the correspondent authors by email with appropriate justification.
